# Somatic Mutations in *HER2* and Implications for Current Treatment Paradigms in *HER2*-Positive Breast Cancer

**DOI:** 10.1155/2020/6375956

**Published:** 2020-03-07

**Authors:** Maria Gaibar, Laura Beltrán, Alicia Romero-Lorca, Ana Fernández-Santander, Apolonia Novillo

**Affiliations:** Faculty of Biomedical Sciences and Health, Universidad Europea de Madrid, C/Tajo, S/N, 28670 Villaviciosa de Odón, Madrid, Spain

## Abstract

In one of every four or five cases of breast cancer, the human epidermal growth factor receptor-2 (*HER2*) gene is overexpressed. These carcinomas are known as HER2-positive. HER2 overexpression is linked to an aggressive phenotype and a lower rate of disease-free and overall survival. Drugs such as trastuzumab, pertuzumab, lapatinib, neratinib, and the more recent afatinib target the deregulation of HER2 expression. Some authors have attributed somatic mutations in HER2, a role in resistance to anti-HER2 therapy as differential regulation of HER2 has been observed among patients. Recently, studies in metastatic ER + tumors suggest that some HER2 mutations emerge as a mechanism of acquired resistance to endocrine therapy. In an effort to identify possible biomarkers of the efficacy of anti-HER2 therapy, we here review the known single-nucleotide polymorphisms (SNPs) of the HER2 gene found in HER2-positive breast cancer patients and their relationship with clinical outcomes. Information was recompiled on 11 somatic HER2 SNPs. Seven polymorphisms are located in the tyrosine kinase domain region of the gene contrasting with the low number of mutations found in extracellular and transmembrane areas. HER2-positive patients carrying S310F, S310Y, R678Q, D769H, or I767M mutations seem good candidates for anti-HER2 therapy as they show favorable outcomes and a good response to current pharmacological treatments. Carrying the L755S or D769Y mutation could also confer benefits when receiving neratinib or afatinib. By contrast, patients with mutations L755S, V842I, K753I, or D769Y do not seem to benefit from trastuzumab. Resistance to lapatinib has been reported in patients with L755S, V842I, and K753I. These data suggest that exploring HER2 SNPs in each patient could help individualize anti-HER2 therapies. Advances in our understanding of the genetics of the HER2 gene and its relations with the efficacy of anti-HER2 treatments are needed to improve the outcomes of patients with this aggressive breast cancer.

## 1. Introduction

Breast cancer is the most common cancer type worldwide and is considered a heterogeneous genomic disease in terms of molecular markers, prognosis, and treatments [1, 2]. At the molecular level, at least five clinical subtypes have been defined: hormone receptor-positive (luminal A and luminal B), human epidermal growth factor receptor-2 (HER2-positive), basal-like, normal-like, and triple-negative breast cancer (TNBC) [2–4]. Based on this classification, the oncologist is able to prescribe the best endocrine therapy, chemotherapy (alone or combined), and/or HER2-targeted therapy. About 20–25% of all breast cancers overexpress human epidermal growth factor receptor-2 (HER2) and are referred to as HER2-positive. HER2 overexpression is linked to an aggressive phenotype resulting in reduced disease-free and overall survival compared with other breast cancer subtypes, and different strategies have been developed to try to block this receptor [5–9]. According to clinical data, HER2-targeted therapy significantly improves the survival of breast cancer patients showing HER2 overexpression. However, recent data suggest the presence of oncogenic mutations in *HER2* affects clinical outcome in HER2-positive breast cancer patients [10].

**Figure 1:**
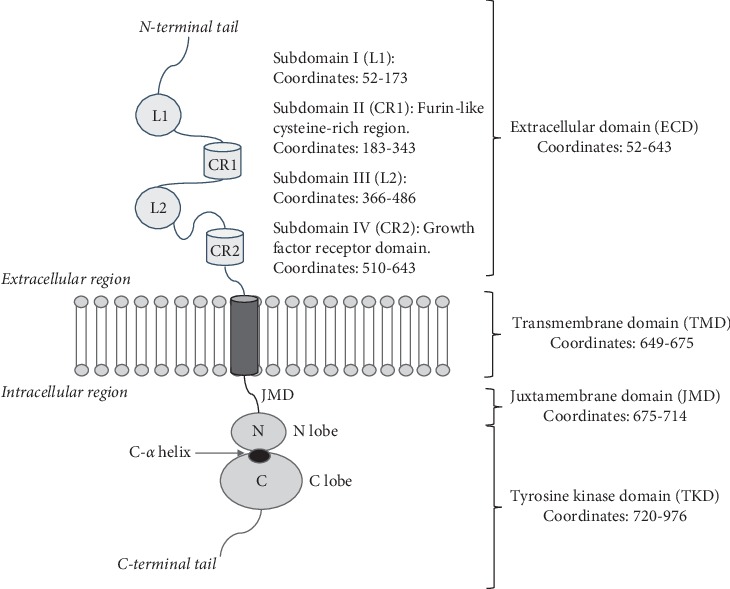
Structural domains of HER2 protein.

**Figure 2:**
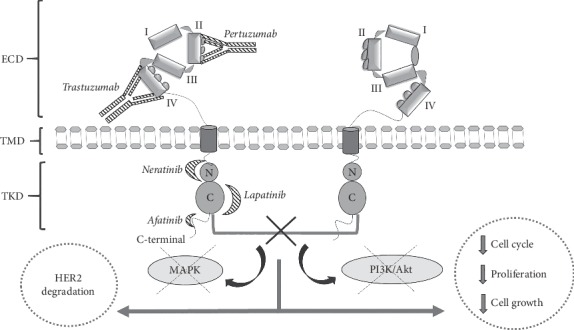
The mechanism of action of different drugs (italics and striped) on HER receptor signaling pathways. HER: human epidermal growth factor receptor; MAPK: mitogen-activated protein kinase; PI3K: phosphatidylinositol 3-kinase; Akt: serine/threonine kinase Akt, also known as PKB (protein kinase B); ECD: extracellular domain; TMD: transmembrane domain; TKD: tyrosine kinase domain.

In 1983, the receptor tyrosine kinase 2 gene (*ERBB2* or newly named *HER2*) was cloned [11]. This gene is located on the short arm of chromosome 17 and its product is the glycoprotein, HER2, which has several functional domains (Figure 1) that resemble those of other members of the tyrosine kinase family (HER1, HER3, and HER4): an extracellular domain (ECD, containing four subdomains), a transmembrane domain (TMD), an intracellular region that consists of a juxtamembrane domain (JMD), and a tyrosine kinase domain (TKD) [12]. HER2 is an atypical member of the ERBB family because it has no known ligand and its ECD constitutively adopts an open conformation [13]. This has led several authors to suggest a role of HER2 as coreceptor [14]. HER2 preferentially heterodimerizes with ligand bound untethered (open) HER3 or with HER4 and HER1, thereby affecting the downstream signaling of these receptors. In overexpressing cells, HER2 forms homodimers that are capable of signaling [13, 15, 16]. HER2 promotes oncogenic signaling by modulating the expression and activity of proteins controlling cell proliferation, differentiation, death, migration, and angiogenesis, activating specific PI3K/Akt (phosphatidylinositol 3-kinase/Akt, also known as PKB, protein kinase B) and MAPK (mitogen-activated protein kinase) pathways (Figure 2). Unlike other ERBB receptors, HER2 remains on the cell surface for prolonged periods after being activated to signal, which contributes to its ability to transform cells when overexpressed. New findings in breast cancer cells indicate that plasma membrane calcium ATPase2 (PMCA2) is vital for the localization of HER2 and its partners, EGFR and HER3, to activate membrane signaling domains contributing to HER2's ability to transform cells when overexpressed and prevent HER2 internalization after receptor stimulation and it sustains downstream signal transduction. This means that targeting PMCA2-HER2 interactions could be a new therapeutic approach [17]. Recently, HER2 and the cannabinoid receptor CB_2_R have been described to physically interact. In effect, the expression of heteromers (HER2-CB_2_R) has been correlated with a poor prognosis, while their disruption promotes an antitumor response suggesting these heteromers could be used as therapeutic targets and prognostic tools in HER2-positive breast cancer [18].

*HER2* gene amplification, or protein overexpression, is still considered a major mechanism of HER2-driven tumorigenesis and is used as a main predictive biomarker to identify patients who might benefit from therapy with anti-HER2 agents. There are, thus, many different cancer drugs approved by the US Food and Drug Administration (FDA) that target the deregulation of HER2, including monoclonal antibodies, antibody-drug conjugates, and small-molecule TKIs (tyrosine kinase inhibitors), such as trastuzumab, pertuzumab, lapatinib, trastuzumab-emtansine (T-DM1), and neratinib [19–21], as well as others under investigation such as afatinib [7, 22–25] (Table 1). Molecular studies have shown that HER2-positive breast cancers are heterogeneous and that the different tumors may be classified as HER2-enriched or luminal molecular subtypes based on estrogen receptor expression (ER), with implications in their response to targeted therapies [26]. Furthermore, *HER2* mutations are identified in 4% of breast cancer patients; these mutations are independently associated with HER2 amplification status, occurring in both hormone receptor (HR)-positive/HER2-negative and HER2-positive [21, 27–30]. Some authors suggest that the prevalence of *HER2* mutations changes according to certain histological subtypes in breast cancer [21, 27, 31].

Recently, data from preclinical and clinical studies have attributed somatic mutations in *HER2*, a role in the constitutive expression [31–33] or differential regulation of HER2 that leads to resistance (primary or acquired) to anti-HER2 therapy and endocrine therapy [4, 6, 10, 34–36]. Such mutations therefore undermine the clinical benefits of HER2-targeted treatment in HER2-positive breast cancer patients. Besides, different mutations in *HER2* have been found in several tumors although their role in tumorigenesis is not fully understood. To assess the possible clinical implications of HER2 mutations in HER2-positive breast cancer patients, we here review the spectrum of single nucleotide polymorphisms (SNPs) produced in the *HER2* gene. Our working hypothesis was that recurrent mutations in specific HER2 domains in these patients could be good biomarkers of the efficacy of anti-HER2 therapy.

## 2. Methods

To identify mutations in the *HER2* gene in HER2-positive breast cancer patients, two databases were searched: cBioPortal [37] and COSMIC [38]. These websites provide information regarding the largest number of studies and *HER2* mutations across different cancer types. To identify mutations reoccurring in HER2-positive breast cancer, the following keywords were used: HER2+ BREAST CANCER, ER-HER2+ BREAST CANCER, and ER-PR-HER2+ BREAST CANCER. In both databases, mutations were observed at similar frequencies. To obtain functional data for the different mutations, we also undertook a PubMed [39] search for articles written in English using the keywords: BREAST CANCER, CANCER RISK, HER2/ERBB2, HER2 POSITIVE, HER2-TYROSINE KINASE DOMAIN, HER2, HER2-TRANSMEMBRANE DOMAIN, HER2-EXTRACELLULAR DOMAIN, and HER2 MUTATIONS.

### 2.1. Mutations in *HER2* Gene in Different Breast Cancer Histologies

Mutations in the ERBB2 receptor described in this study according to the tumor type were found in invasive lobular carcinoma (ILC), invasive ductal carcinoma (IDC), and mixed ductal and lobular carcinoma (MDLC) (Table 2). There is variability in the distribution of the different mutations depending on the specific histology of the breast cancer type. Seven of the eleven mutations were present in both types of carcinomas or even in mixed carcinomas (MDLC); however, some of these mutations are mainly found in IDC or others in ILC (Table 2). Thus, mutations located mainly in IDC were D769H, V842I, K753E, R678Q, and S310F I655V. In the other side, mutations more prevalent in ILC were L755S, V777L, D769Y, and S310Y. Previous studies suggest that *HER2* mutations are enriched in certain histological subtypes, as example, some authors have indicated that invasive lobular breast cancer (ILC), which composes about 15% of estrogen receptor- (ER-) positive subtype, the prevalence of *HER2* mutations is higher (cBioPortal-21, 27, 56-ILC). No quantitative analysis of the presence of specific mutations according to tumor type has been performed in this study, but the *HER2* mutations described here located in IDC and ILC are in agreement with other studies [27, 31, 56, 57]. Interestingly, *in silico* analysis suggests that some *HER2* mutations are enriched in primary ILC and their detection represents an actionable strategy with the potential to improve patient outcomes with estrogen receptor-positive, ERBB2 nonamplified primary lobular [27]. Overall, more quantitative studies are needed for the identification of co-occurring and mutually exclusive *HER2* mutations according to histology subtype in order to identify patient that could potentially be targeted with HER2-directed therapies.

### 2.2. Mutations in the Tyrosine Kinase Domain

Most mutations in the *HER2* gene have been detected in exons 19 and 20 of the tyrosine kinase (TK) domain, at the C-*α* helix position of the protein [34] (Table 2). Several authors propose that mutations in this domain could be an alternative mechanism to HER2 activation and affect sensitivity to anti-HER2 therapy, as an acquired resistance mechanism to this form of therapy. The TKD mutations described to date in HER2+ breast cancer promote the activation of the functionality of the protein and increase the oncogenicity of HER2, besides inducing the phosphorylation of other cell signaling proteins [28, 34] (Table 2). This is because this domain contains the ATP binding site and its mutations are related to the enhanced phosphorylation of receptors HER2, HER3, and HER1, which causes receptor HER2 dimerization along with protein ERK (extracellular signal-regulated kinase) and AKT phosphorylation, with consequent activation of the PI3K/Akt and MAPK pathways, finally enhancing cell proliferation and angiogenesis (Figure 2). The binding site of ATP with the receptor protein forms a conformational structure with other important structures such as phosphate activation and binding loops, which could be affected by such modifications. Missense substitutions usually occur at the C-*α* helix, which is essential for HER2 protein activation. These alterations can promote tumorigenesis and phosphorylation of signaling proteins such as phospholipases *γ*C1 and C*γ* (PLC*γ*) MAPK. Many of these activating mutations have proved resistant to anti-HER2, such as those found at codons 755 or 798 [34].

Most authors have described the appearance of both intrinsic and acquired resistance to trastuzumab therapy in mutations L755S, V777L, D769Y, and K753E [32, 40, 42, 44, 45]. As these mutations are not located close to the drug's binding target, it seems that rather than blocking receptor binding of the drug, they affect resistance to its effects by increasing kinase activity and activation of the protein's oncogenic signaling pathways, independently of drug binding. All these mutations as well as D769H share the feature of sensitivity to the actions of the irreversible TK inhibitor, neratinib [28, 30, 35, 41, 42, 45]. This could be explained by the greater strength of interactions produced between this drug and the ATP-binding site. This response offers a good treatment option for patients who may have developed resistance to first-line treatments for HER2+ breast cancer. Most authors agree that resistance to lapatinib, both intrinsic and acquired, appears in L755S, D769Y, V842I, and K753E [41, 42, 44]. This indicates the importance of the electrostatic interactions that occur at the ATP binding site close to these residues. Moreover, depending on the changes produced by the amino acid substitutions, a protein conformation may arise that promotes either the active state of HER2's kinase domain impairing proper drug binding or this binding increases sensitivity toward the drug.

The L755S mutation is the most common in *HER2* gene [41] and is considered a hotspot mutation [58]. The protein's codon 755 seems to be strongly involved in activating HER2 receptor kinase, which leads to the potentiated activity of the PI3K/Akt and MAPK signaling pathways, giving rise to enhanced cell proliferation and angiogenesis. In preclinical trials, this mutation has been associated with resistance to lapatinib treatment through reactivation of HER2 signaling in HER2+ breast cancer models in which the gene is overexpressed [43, 44]. In *in vitro* models, it was observed that cells with this mutation were resistant to treatment with lapatinib + trastuzumab, but also to trastuzumab + pertuzumab treatment [32, 40, 41]. As this mutation induces resistance to trastuzumab alone or in combination with pertuzumab, despite its location far from the drugs' binding sites on the receptor, it could be that kinase activity is so enhanced that it is able to continue signaling despite the nondimerization of the receptor after the binding of these drugs [40, 41, 44, 59]. Resistance to lapatinib can be explained by the fact that Leu 755 participates in hydrophobic interactions with the C-*α* helix of the TKD in the active state of HER2, while in the inactive form, L755 is found far from this helix [43]. The L755S polymorphism induces the appearance of polar interactions that stabilize the active form; this would help explain resistance to lapatinib, which only binds to the inactive conformation of HER2 [43]. This resistance could be addressed with irreversible HER1 and HER2 inhibitors such as neratinib, which has proven effective in patients with this mutation [41]. In effect, *in vitro* studies have shown the sensitivity of cells with the L755S mutation to afatinib plus neratinib [41, 44]. Besides intrinsic resistance, mutation L755S has been associated with resistance acquired to trastuzumab therapy in breast cancer. It appears in 7.59% of patients receiving prior trastuzumab treatment. Further, it has been reported to occur in 3 out of every 18 patients with metastasis but not those with primary tumors [41].

Mutation V777L is also considered hotspot [58]. Residue V777L, located in exon 20 (at the C-terminal tail of the C-*α* helix), is involved in TK activity. This activating mutation promotes the TK activity of HER2, increasing the phosphorylation of signaling proteins such as HER2, HER3, EGFR, and ERK, and the transformation of breast epithelial cells [29, 33, 40, 45, 60]. This mutation causes transcriptional activation in most tumors affected by this mutation, which usually occurs independently of *HER2* gene activation [60]. In effect, cases have been described in breast cancer cell lines in which increased endogenous expression levels of HER2 V777L activated signal transduction pathways, but this did not significantly increase tumor growth [61]. The effects of V777L seem enhanced by mutations in the PIK3CA gene given that, in the presence of mutation PIK3CA E545K, V777L gives rise to enhanced interaction between p58 and HER3. This suggests that reverse mutations of the *HER2* gene could require other genetic alterations to promote cellular transformation and enhance interactions between signaling partners [31]. This mutation has been associated with the intrinsic development of trastuzumab resistance [45]. Although the mutation has been associated in some preclinical studies with a diminished response to lapatinib, afatinib, and neratinib, several studies have shown reduced tumor growth and signaling activity in tumors with the V777L mutation treated with lapatinib [44, 45]. A response has been observed to combined treatment with neratinib and other drugs in patients with ER + V777L breast carcinoma [35]. No cases relating this mutation to the response to pertuzumab have been described. Considering that this last drug, as does trastuzumab, binds to the extracellular domain of the protein and that resistance to trastuzumab has been described, we would expect pertuzumab to neither elicit a good response in patients with this mutation. As occurs with the L755S mutation, *HER2* V777L shows strong activation of the receptor's kinase that could preserve its signaling activity even with trastuzumab and pertuzumab bound to the extracellular domain of the protein.

Interestingly, V777L and L755S mutants have been characterized using molecular dynamics simulations and *in vitro* studies in Ba/F3 cells expressing these mutants, showing that these mutants have a larger binding pocket volumes and therefore are more sensitive to tyrosine kinase inhibitors (TKIs) of quinazoline (afatinib and poziotinib) and indole (osimertinib and nazartinib) groups. Furthermore, in preclinical models, poziotinib upregulates HER2 cell surface expression and potentiates the activity of T-DM1, inducing a complete tumor regression with combination treatment [62]. The authors of this study suggest that poziotinib in combination with T-DMI could be a good candidate treatment for not only non-small cell lung cancer; in fact, one ongoing trial in phase II is studying the efficacy of poziotinib in metastatic breast cancer harboring *HER2* mutations [21, 62]. Overall, more clinical studies are needed to test the efficacy of poziotinib in combination with T-DMI in breast cancer to rule out differences in tumor type-specific sensitivities to the same pharmacological product. In SUMMIT trial, neratinib was most effective in breast cancer patients, with patients containing L755S and V777L [33], but the same mutations were associated with resistance in other cancer types, suggesting that more research is needed to identify the mechanism involved in tumor-type-specific sensitivities.

Recently, using isogenic knock-in *HER2* mutations in ER + MCF7 cells and xenografts, two activating *HER2* mutations located in the kinase domain (L755S and V777L) emerged as resistance to anti-ER therapy progression [35]. These findings are corroborated by other authors, and the same mutations have been identified in metastatic biopsies of eight patients with ER + metastatic breast cancer (MBC), as mutations that were acquired under the selective pressure of ER-directed therapy such as aromatase inhibitors [36]. The same authors demonstrated that the resistance to ER-directed therapy was overcome by combining fulvestrant with the irreversible HER2 kinase inhibitor neratinib. These data suggest that the prevalence of *HER2* mutations might increase in metastatic ER+ breast cancer treated with anti-ER therapy, and these mutations are a distinct mechanism of acquired resistance to ER-directed therapy in metastatic breast cancer that could be solved by the treatment with an irreversible HER2 inhibitor. Overall, these data suggest that patients with ER+/HER2 mutations would benefit from HER2-targeted therapies in combination with hormonal therapy. If ongoing clinical trials confirm these results, new approaches could be adopted in order to promote a better response in patients with ER + MBC, and one of these strategies could be to identify HER2-mutant-resistant clones to ER-directed therapy [36].

Mutation V842I has been detected in various types of tumor tissue. This is also an activating mutation associated with *HER2* gene amplification and increased phosphorylation of different signaling proteins [28] and also represents a hotspot in *HER2* [58].

The effects of V842I on the response to anti-HER2 therapy in patients with HER2+ breast cancer have not been yet explored. Some *in vitro* studies indicate the resistance to trastuzumab and lapatinib of cell lines with this mutation [44]. This mutation is the most common mutation in colorectal cancers, and *in vitro* studies have shown that this mutant was not sensitive to neratinib [62]. However, given its recurrent expression in different tumor tissues and its association with amplification of the gene, studies are warranted to clarify its impact on the receptor's kinase activity.

The nonsynonymous mutations D769Y and D769H are among the most frequent somatic mutations of the *HER2* gene. They are located in exon 19, at position 769 of the TK domain, which is important for ATP-HER2 binding [29]. Both mutations have been characterized as activators in mammary epithelium cell lines, and *in vivo* studies have revealed neratinib as effective at blocking tumor growth in HER2+ breast carcinomas with these mutations [28, 42].

Cases have been described of xenografts acquiring the D769Y mutation following treatment with trastuzumab, along with their subsequent resistance to trastuzumab and lapatinib, suggesting its possible role in acquired resistance to anti-HER2 therapy [42]. In mutation D769Y, the change from aspartic acid to tyrosine could lead to changes in electrostatic interactions, due to the substitution of a negatively charged acid side chain at physiological pH with the capacity to form hydrogen bridges and bind phosphate groups. As this mutation occurs at an important position for ATP binding to the receptor, this change could benefit this binding and thus diminish the impacts of lapatinib and neratinib therapy, whose mechanism of action is to impair this binding of ATP to HER2 [42]. The D769Y mutation promotes the phosphorylation of HER2, EGFR, HER3, and ERK and transformation of mammary epithelial cells. Cell lines with this mutation display sensitivity to neratinib, in smaller measure to lapatinib and resistance to trastuzumab [42], although Nagano et al. recently described sensitivity to lapatinib and afatinib in *in vitro* studies [44]. Some authors report that loss of the acid side chain or addition of an aromatic ring to amino acid 769 could increase HER2's TK activity due to dimeric interactions between the kinase domains of HER2 and HER3. Mutations D769H/Y may enhance hydrophobic contacts and heterodimerization of HER2. Besides, the D769H alteration could lead to activation within the HER2 monomer, adding hydrogen bonds to its own activation A-loop [46, 54].

Mutation K753E leads to a shift in charge of the amino acid's side chain, which goes from being basic to acidic, thus possibly affecting the electrostatic interactions of the protein. Several authors have related this mutation with lapatinib resistance, and this could be attributed to its close proximity with the L755S mutation which confers resistance to this drug [32, 41]. Recently, the effect of this mutation has been observed in cell lines overexpressing HER2 K753E. In HER2 K753E mutant cells resistant to lapatinib, a greater affinity of the drug for the HER2 protein was observed compared to wild-type cells and other variants. This reveals that resistance to this drug is unrelated to a lack of binding to its target [63]. It has also been related to resistance to trastuzumab and appears in 2 out of every 18 patients with metastasis [32]. While cell lines that show this mutation are resistant to lapatinib, they are sensitive to neratinib, which could benefit patients developing resistance to trastuzumab therapy [41].

Following trastuzumab therapy, the appearance of K753E and L755S mutants could suggest their potential role as drivers of developing trastuzumab resistance during HER2+ tumor progression [32].

Mutation I767M is a hotspot in gene *HER2* [58] identified in patients with HER2+ breast cancer [54]. Its expression has been examined *in vitro* in HER2-overexpressing mammary cell lines and in HER2-negative cultures. In the former cells, the presence of this mutation along with mutations in the genes *PIK3CA* and *TP53* conferred a significant growth benefit over cells with the wild-type HER2 gene. Further, both the mutant and wild-type protein featured similar AKT and MAPK signaling levels, although the AKT pathway remained active over time for longer in the cells expressing HER2 I767M [47]. *In vitro* studies conducted by Nagano et al. [44] indicate the sensitivity of I767M to therapy with both TK inhibitors (lapatinib, neratinib, and afatinib) and the monoclonal antibody trastuzumab.

According to the data from COSMIC and cBioPortal, while other mutations in this kinase domain have been described (i.e., V797A, D808E, D873G, and M889I), there are still no data regarding their role in HER2+ breast cancer.

### 2.3. Mutations in the Juxtamembrane Domain

The juxtamembrane domain, containing 39 amino acids (Figure 1), is involved in receptor dimerization and stability. Several authors have described reoccurring mutations in this domain with a functional activating effect in different cancer types [10]. However, these studies do not specify if these mutations occur in patients with HER2-positive breast cancer. In our search of mutations in the COSMIC and cBioPortal databases, we found two mutations, R678Q and V697L, present in HER2-positive breast carcinoma. *In vitro* studies indicate that R678Q is an activating mutation that confers sensitivity towards treatment with trastuzumab, lapatinib, afatinib, and neratinib (Table 2) [10, 33, 44] and has been classified as a hotspot [64]. No functional data exist for mutation V697L, but it has been described as a mutational hotspot and data available for other cancer types suggest its oncogenic effect.

### 2.4. Mutations in the Transmembrane Domain

The transmembrane domain of receptor HER2 (aa 649–675, Figure 1) plays an active role in its dimerization with the consequent activation of kinase activity and promotion of the signaling pathways responsible for tumor cell growth. Recently, recurrent mutations have been identified with an activating effect in different cancers [10]. However, the data sources COSMIC and cBioPortal reveal that no mutations in this domain occur in HER2-positive breast cancer. In the present study, we identified only one mutation, I655V (Table 2), in HER2-positive patients, which, according to Fleishman et al. [65], involves an altered receptor conformation that renders it a constitutively activated state, promoting the homodimerization and autophosphorylation of HER2 and activation of the TK domain [65]. Singla et al. [49] found, among patients in Indian hospitals, a positive significant association between *HER2* I655V and the susceptibility of developing breast cancer, while other authors have detected negative correlation when examining patients in Brazil [66]. A meta-analysis conducted in 2019 [67] revealed the impacts of ethnicity on the association between mutation *HER2* I655V and breast cancer risk, observing positive correlation in Asia and Africa but not the other continents.

The relationship between this mutation and the response to trastuzumab-containing chemotherapy in HER2-positive breast cancer patients has been examined, yet results have been contradictory. In some studies, disease-free survival (DFS) and delayed DFS (DDFS) were improved in patients with this mutation and the genotypes *HER2* Val/Val or Val/Ile compared to the genotype Ile/Ile [48]. On the contrary, Furrer et al. [52] noted a worse response to trastuzumab-containing chemotherapy, while other studies have found no correlation [49–51]. These results preclude establishing a clear relationship between this polymorphism and the development of resistance to treatment with trastuzumab. The effect of trastuzumab and other antibodies may be limited in tumors that show mutations in the TMD, as HER2 dimerization seems stable despite trastuzumab binding to its extracellular domain. Neratinib, which binds to the HER2 receptor's kinase domain and inhibits its phosphorylation and activity, can exert antitumor effects irrespective of the domain affected by mutations and has an impact on this *HER2* I655V mutation in different lung cancer cell lines [68].

Something similar occurs with the risk of cardiotoxicity induced by trastuzumab. Despite initial proposals that being a carrier of the mutant allele, genotypes *HER2* Val/Val or Val/Ile, was a possible predictor of this adverse effect of the drug [50], this relation could not be later confirmed [53]. Some authors have suggested that carrying this mutation only affects the survival of patients with breast cancer who overexpress the *HER2* gene [48]. To date, the possible effect of this mutation on the actions of other HER2-target drugs such as pertuzumab, neratinib, afatinib, or lapatinib has not been addressed.

### 2.5. Mutations in the Extracellular Domain

The extracellular domain is composed of four subdomains involved in dimerization of the receptor and thus in its activation. Several mutations in the ECD domain have been described in patients with HER2-positive breast cancer both in PubMed and the databases COSMIC and cBioPortal, as described below. The most common mutations in the ECD of the HER2 receptor, S310F and S310Y, corresponding to the gene hotspot (Table 2) [64], have been related to the increased dimerization of the receptor, kinase activation, and malignant cell transformation. Both mutations appear to have a homologous effect. Of the two, S310F has been most studied in different tumor tissues (both HER2-positive breast and HER2-negative lobular breast, lung, colorectal, ovarian, bladder, micropapillary urothelial, and endometrial) [29, 33, 54, 68–70] while S310Y has been more commonly associated with pulmonary adenocarcinoma while it has been also found in HER2-positive and HER2-negative breast cancer [29, 33, 55, 71]. The fact that mutations in this position are present in different cancers suggests it could be an oncogenic mutation [72]. They are therefore mutations that activate HER2 protein via elevated phosphorylation of the C-terminal tail, as is the case of mutation S310F/Y, or inducing covalent dimerization sustained by intermolecular disulfide bridges [73], as in the case of mutations G309E/A, only described in HER2-negative breast cancer and other cancers [28, 73]. In the presence of an S310F/Y mutation, it has been noted that protein HER2 seems more sensitive to anti-HER2 therapy containing neratinib and possibly trastuzumab in patients with HER2+ breast cancer [33, 54, 55]. Accordingly, in cells featuring ECD mutations, trastuzumab may bind to this region and prevent homodimerization and activation of the receptor. However, because of the constant activation of the TKD in tumors with mutations at this domain, the antiproliferative effects of monoclonal antibodies may be limited despite inhibiting dimerization [74]. The effects of these mutations on the response to pertuzumab and trastuzumab have been investigated recently using *in vitro* 5637 culture cells and single-molecule interaction analyses using TIRF microscopy [74]. The overexpressed S310F as well as G309A, G309E, and S310Y HER2 mutants reacted to trastuzumab, but S310F mutant did not react to pertuzumab along with S310Y or G309E mutants. Thereafter, authors tested the effects of trastuzumab and pertuzumab using both wild-type HER2 and S310F mutant. In this case, trastuzumab did not inhibit the activation of the HER2 receptor, suggesting that the S310F HER2 mutant did not form homodimers or heterodimers with wild-type HER2. Because pertuzumab did not inhibit the phosphorylation of HER2 while it bound to wild-type HER2, EGFR-mediated phosphorylation is expected to occur on the S310F mutant; therefore, trastuzumab in combination with pertuzumab is not effective [74]. This residue is located close to one of the key residues, K311, for receptor-antibody binding whose replacement with alanine, via targeted mutagenesis, leads to a drastic reduction in the response to this drug in cells expressing the mutation. Amino acid substitutions in these residues could provoke changes in electrostatic interactions or even give rise to a stearic impediment possibly affecting pertuzumab binding to the HER2 receptor [69].

Other less frequent mutations in the ECD have been described, such as R190Q, P523S, and Q548R, in patients with breast cancer without specifying HER2 amplification, in which no relationship has been found between the mutations and prognosis [75]. Even rarer are L313I and R456C, observed in two patients with HER2+ breast cancer effectively treated with neratinib [42].

The COSMIC and cBioPortal databases also describe other mutations in this domain about which there are no published data for HER2-positive breast cancer: A37T, P232S, D277H (also described in bladder cancer, enhances its activation together with S310F, de Martino et al. [76]), T297I, E405D, and H470Q.

### 2.6. Beyond HER2+ Breast Cancer: Lessons from Clinical Data from the Use of HER2-Directed Therapy against HER2 Mutant Cancers

Around sixteen clinical trials investigating the efficacy of HER2-directed therapy in HER2 mutant cancers are currently active [21]. Four phase II studies are studying the efficacy of different pharmacological products (afatinib, neratinib plus trastuzumab, poziotinib, and pyrotinib) in different types of metastatic HER2 nonamplified but with HER2 mutant breast cancer. There are a relatively large number of pharmacological approaches for breast cancer carrying HER2 mutants. In part, we have reached this situation because activating mutations for *HER2* have been shown to be largely dependent on tumor histology and have shown different clinical responses. Some mutations are sensitive in a specific type of cancer and in others could be associated with resistance, suggesting that there may be other mechanisms specific with the tumor that requires further research.

**Figure 3:**
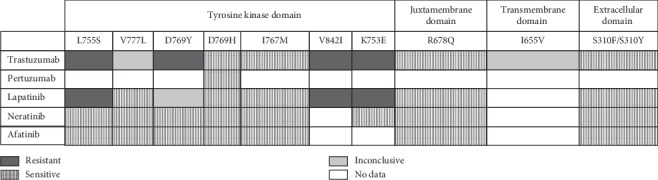
Pharmacological impacts of the SNPs reviewed in this study. The sensitivity of HER2 mutants to different drugs used as anti-HER2 therapy is shown. The pharmacological products have different levels of activity against mutant HER2+ proteins *in vitro.* When data from *in vivo* studies (xenotransplant and/or breast cancer patients) were available, they were considered for the analysis. Furthermore, some mutants that have been described to be sensitive to specific inhibitors in preclinical analyses were instead found to be resistant to the same drugs; in this case, we have indicated this information as inconclusive data.

The focus of this review is to assess the possible clinical implications of *HER2* mutations in HER2-positive breast cancer patients; in this study, we have described that the most prevalent mutations found in *HER2* gene in HER2-positive breast cancer (Table 2) are present also in HER2-negative breast cancer [28, 29, 32, 34, 57]. We would like to address, using clinical data available, if HER2-negative patients with *HER2* somatic mutations are potentially good candidates for HER2-directed therapy. The clinical data available have been reviewed by Cocco et al. [21] and are summarized in Table 3. The first patient diagnosed with triple-negative breast cancer, carrying two HER mutations (V777L and S310F), respond to lapatinib and trastuzumab-based therapies during 6 months. A second case diagnosed with ER + HER-negative breast cancer, carrying a HER2 S310F mutation, was treated during 12 months with the combination of trastuzumab, pertuzumab, and fulvestrant. An additional case with ER+, HER2-negative metastatic breast cancer with HER L755S mutation was treated with neratinib monotherapy experiencing improvement in symptoms and tumor markers. Another case described a *HER2* (D769H) mutant with metastatic HER2-negative breast who achieved a partial response with trastuzumab, pertuzumab, and chemotherapy (Table 3). These clinical data are in agreement with the pharmacological profile of the SNPs of HER2 reviewed in this study (Figure 3). In the phase II MutHER trial, the activity of neratinib in HER2 mutant nonamplified metastatic breast cancer was investigated (Table 3); the patients obtained clinical benefit over 24 months [77]. A case report was a HER2-negative breast cancer patient with two detected mutations in ERBB2 (S310F and D769Y mutations) who benefited from lapatinib combined with endocrine therapies [78]. Based on clinical data available for HER2-negative breast cancer patients (Table 3), functional activating HER2 mutations, V777L, L755S, S310F, D769H/Y, and V842I, may similarly confer sensitivity to HER2-directed pharmacological products. Furthermore, a high number of HER2-mutant tumors are also ER+, and as discussed before, the most effective treatment will be combining fulvestrant with the irreversible inhibitor neratinib [21, 35]. Overall, HER2-negative breast cancer patients carrying the above mutations can benefit from HER2-targeted therapy; this is in agreement with data previously published by other authors [32, 78].

### 2.7. Conclusions and Future Perspectives

Research and clinical studies have shown that HER2 overexpression/amplification is associated with poor survival in breast cancer patients. Further, *in vitro* and *in vivo* studies indicate that the presence of somatic HER2 mutations could influence the clinical outcome of HER2-positive patients under currently approved treatments (Table 1). New findings in breast cancer cells suggest that HER2 could interact physically with PMCA2 and the cannabinoid receptor CB_2_R. Hence, targeting these heteromers could be a new therapeutic option and prognostic tool in HER2-positive breast cancer. Recently, *in vitro* and *in vivo* studies in metastatic ER + tumors suggest that some HER2 mutations emerge as a mechanism of acquired resistance to endocrine therapy opening new options of treatments in patients with ER + MBC.

**Figure 4:**
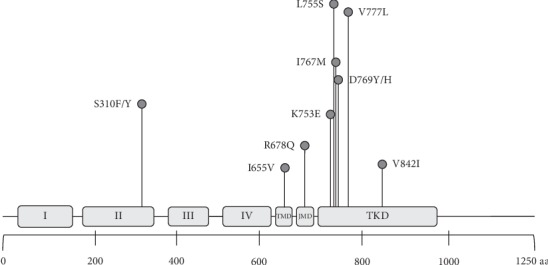
Schematic diagram of HER2 protein with the locations of the SNPs reviewed in this study found in HER2-positive breast cancer patients. Domains I, II, III, and IV belong to the extracellular domain (ECD); TMD: transmembrane domain; JMD: juxtamembrane domain; TKD: tyrosine kinase domain.

In this review, we identify the more prevalent somatic HER2 SNP mutations appearing in HER2-positive breast cancer patients and summarize their possible implications for current HER2-targeted therapy (Figure 4). We found that somatic HER2 mutations occur in low frequency in HER2-positive breast cancer patients. In total, 11 somatic mutations have been identified, and according to information available from *in vitro* and *in vivo* studies, 9/11 are classified as oncogenic and hotspot (see Table 2), and several authors have identified the presence of these mutations also in HER2-negative breast cancer patients. For two mutations, I767M and K753E, there is insufficient information so far to classify them as oncogenic and/or hotspot. In HER2-positive tumors, the TKD harbored the higher number of somatic mutations (7/11), contrasting with the low number of mutations found in the extracellular and transmembrane domains. The relevance of some mutations identified in this study requires further investigation.

For the reviewed somatic HER2 mutations, no sensitivity or resistance data are available for pertuzumab, with the exception of mutation D769H. For some mutations, available data are inconclusive requiring more functional studies. HER2-positive patients carrying S310F, S310Y, R678Q, D769H, I767M, or V777L emerged as potentially good candidates for HER2-targeted therapy and could have a favorable outcome because of sensitivity to current pharmacological treatments with the exception of inconclusive data for the impacts of trastuzumab in V777L (Figure 3). Patients with L755S or D769Y might also benefit from neratinib or afatinib treatment. In contrast, patients with the somatic mutations L755S, V842I, K753I, or D769Y do not seem to benefit from trastuzumab. Similar negative results have been observed for lapatinib in patients carrying the L755S, V842I, and K753I mutations. L755S and V777L mutations emerge as a distinct mechanism of acquired resistance to anti-ER therapies in ER+ metastatic breast cancer that was overcome by combining fulvestrant with the irreversible inhibitor neratinib. Furthermore, patients with metastatic breast cancer HER2+ with L755S and V777L could benefit of treatment with a new TKI, poziotinib, that is in phase II of clinical trials. Clinical studies suggest that HER2-negative breast cancer patients carrying the *HER2* mutations reviewed here can benefit from HER2-targeted therapy. In future studies, different combinations of mutations in patients and their treatment with different combinations of drugs need to be considered.

## Figures and Tables

**Table 1:** Current therapeutic approaches targeting HER2 signaling [7, 22–25].

Drug	Molecular target	Molecular mechanism	Treatment options
Lapatinib	TKD ⟶ HER2 and HER1 ATP mechanism of action binding sites	Reversible inhibitor of HER1 and HER2 trans- and autophosphorylation	With metastasis: +capecitabine or letrozole +trastuzumab

Neratinib	TKD ⟶ HER1, HER2, and HER4 ATP binding sites	Irreversible inhibitor of HER1, HER2, and HER4 trans- and autophosphorylation	Adjuvant after trastuzumab treatment

Trastuzumab	Subdomain IV of HER2 ECD	Inhibitor of HER2 homodimerization	First-line anti-HER2 treatment

Ado-trastuzumab emtansine (T-DM1)	Subdomain IV of HER2 ECD	Inhibitor of HER2 homodimerization, cytotoxic action of emtansine	Specific cases after anti-HER2 treatment with trastuzumab

Pertuzumab	Subdomain II of HER2 ECD	Inhibitor of HER2 heterodimerization	Dual therapy: anti-HER2 with trastuzumab + docetaxel/paclitaxel or + capecitabine/vinorelbine

Afatinib	TKD: HER1, HER2, HER4 ATP binding sites	Irreversible inhibitor of HER1, HER2, and HER4 trans- and autophosphorylation	Under research: used as monotherapy in patients with HER2-positive breast cancer showing progression despite trastuzumab treatment. Pending FDA approval

TKD: tyrosine kinase domain; ECD: extracellular domain; HER2: human epidermal growth factor receptor 2.

**Table 2:** Main features and pharmacological implications of the HER2 gene SNPs reviewed in HER2-positive breast cancer patients. ILC: invasive lobular carcinoma; IDC: invasive ductal carcinoma; MDC: mixed ductal and lobular carcinoma. These mutations are found also in HER2-negative breast cancer [28, 29, 32, 34, 77].

Mutation	Exon	Tumor type	Protein domain	Mutation impact	Pharmacological implications	Study design	References
L755S	19	ILC IDC MDC	TKD, C-*α* helix	Activation	Trastuzumab/lapatinib resistance Neratinib/afatinib sensitivity	Breast cancer HER2+ patients, *in vitro* studies	[40, 41]
					Lapatinib resistance	Breast cancer HER2+ patients, *in vitro* studies	[42, 43]
					Trastuzumab resistance Afatinib/neratinib sensitivity	MANO method and xenograft	[44]
					Afatinib/neratinib sensitivity	MANO method and xenograft	[41, 44]

V777L	20	ILC IDC	TKD, C-*α* helix, C-terminal tail	Activation	Trastuzumab resistance Lapatinib/neratinib sensitivity	Breast cancer HER2+ patients	[45]
					Trastuzumab resistance Lapatinib/neratinib/afatinib sensitivity	MANO method and xenograft	[44]
					Trastuzumab + lapatinib sensitivity	Breast cancer HER2+ patient	[31]
					Neratinib sensitivity	Breast cancer HER2+ patient	[35]

D769Y	19	ILC IDC	TKD, C-*α* helix	Activation	Neratinib sensitivity Trastuzumab/lapatinib resistance	Xenograft study	[42]
					Trastuzumab resistance Afatinib/lapatinib/neratinib sensitivity	MANO method and xenograft	[44]

D769H	19	IDC	TKD, C-*α* helix	Activation	Neratinib sensitivity	Breast cancer HER2+ patient	[28]
					Trastuzumab/pertuzumab sensitivity	Breast cancer HER2+ patient	[46]
					Trastuzumab/afatinib/lapatinib/neratinib sensitivity	MANO method and xenograft	[44]

I767M	19	IDC ILC MDLC	TKD, C-*α* helix	Inconclusive	Trastuzumab/lapatinib/afatinib/neratinib sensitivity	*In vitro* breast cell cultures; MANO method; xenotransplant; breast cancer HER2+ patients	[44, 47]

V842I	21	IDC ILC	TKD, c-loop	Activation	Lapatinib/trastuzumab resistance	MANO method and xenograft	[44]

K753E	18	IDC	TKD, C-*α* helix	Likely neutral	Lapatinib/trastuzumab resistance Neratinib sensitivity	Breast cancer HER2+ tumors	[32, 41]

R678Q	17	IDC	JMD	Activation	Trastuzumab/lapatinib/afatinib/ Neratinib sensitivity	MANO method and xenograft	[44]

I655V	16	IDC	TMD	Activation	Trastuzumab sensitivity	Breast cancer HER2+ patients	[48]
					No correlation with trastuzumab efficacy		[49–51]
					Trastuzumab resistance		[52]
					No correlation with trastuzumab-induced cardiotoxicity		[53]
					Correlation with trastuzumab-induced cardiotoxicity		[50]

S310F	8	IDC ILC	ECD, subdomain II, furin-like domain CR1	Activation	Neratinib/trastuzumab sensitivity	Breast cancer HER2+ patients	[33, 54]
					Trastuzumab/lapatinib/afatinib/neratinib sensitivity	MANO method and xenograft	[44]

S310Y	8	ILC	ECD, subdomain II, furin-like domain CR1	Activation	Neratinib/trastuzumab sensitivity	Breast cancer HER2+ patients	[33, 55]
		IDC MDLC			Trastuzumab/lapatinib/afatinib/neratinib sensitivity	MANO method and xenograft	[44]

**Table 3:** Clinical response of HER2 mutant breast tumors to anti-HER2-based therapy.

No. patients	Type of breast cancer	HER2 mutation	Pharmacological treatment	Outcome	Reference
1	Triple-negative	V777L S310F	Lapatinib, trastuzumab	Improvement during 6 months	Reviewed in 21
1	ER+/HER-negative	S310F	Trastuzumab, pertuzumab fulvestrant	Improvement during 12 months	Reviewed in 21
1	ER+/HER-negative	L755S	Neratinib	Improvement during 12 months	Reviewed in 21
1	Metastatic HER2-negative	D769H	Trastuzumab, pertuzumab chemotherapy	Partial response	Reviewed in 21
1	HER2-negative	S310F/V842I	Neratinib	Benefit	[77]
6	HER2-negative	L755S	Neratinib	Benefit	[77]
1	HER2-negative	D769H	Neratinib	Benefit	[77]
1	HER2-negative	p.L755_T759del	Afatinib, trastuzumab	Response	[78]
1	HER2-negative	S310F and D769Y	Lapatinib and endocrine therapy	Response	[78]
